# Aggressive Native Valve *Staphylococcus pasteuri* Infective Endocarditis Complicated by Recurrent Coronary and Cerebrovascular Emboli

**DOI:** 10.1016/j.jaccas.2026.108362

**Published:** 2026-07-15

**Authors:** Riya Sam, Anil Ananthaneni, Kifah Hussain, Juan Pablo Salazar Adum, Mark Lampert, Justin Levisay, Robert Gordon, David Najman, Nikola Dobrilovic

**Affiliations:** a Division of Cardiology, Endeavor Health, Glenview, Illinois, USA; b Division of Cardiology, University of Chicago Pritzker School of Medicine, Chicago, Illinois, USA; c Division of Hematology and Oncology, Louisiana State University Health Sciences, Shreveport, Louisiana, USA; d Division of Cardiology, Northwestern Memorial Hospital, Chicago, Illinois, USA; e Division of Cardiology, Mass General Brigham & Womens Hospital, Boston, Massachusetts, USA; f Division of Cardiothoracic Surgery, Endeavor Health, Glenview, Illinois, USA

**Keywords:** coagulase-negative staphylococci, infective endocarditis, mycotic aneurysm, native valve endocarditis, septic coronary embolism, *Staphylococcus pasteuri*

## Abstract

**Background:**

*Staphylococcus pasteuri* is a rare coagulase-negative staphylococcus that is infrequently identified as a causative agent in infective endocarditis. Its clinical virulence and embolic potential are not well characterized in current literature.

**Case Summary:**

A 57-year-old man with a history of mitral valve prolapse presented with a 2-month history of constitutional symptoms and hematuria. Investigations revealed *S pasteuri* bacteremia and a large mitral valve vegetation with severe regurgitation. His clinical course was marked by recurrent ST-segment elevation myocardial infarctions and mycotic aneurysm, subacute embolic strokes with hemorrhagic transformation. He ultimately underwent successful bioprosthetic mitral valve replacement after neurologic stabilization.

**Discussion:**

This case highlights the aggressive nature of *S pasteuri* in native valve endocarditis. The organism produced large, friable vegetations, leading to a high embolic burden and affecting both coronary and cerebral vasculatures.

**Take-Home Messages:**

*S pasteuri* is a virulent pathogen capable of causing significant endocardial destruction and should not be dismissed as a contaminant. Large vegetations associated with this organism carry an exceptionally high risk for recurrent embolic events, including rare coronary septic embolization. Early multidisciplinary consultation is vital to navigate the timing of surgery when embolic risk and intracranial hemorrhage coexist.

## Visual Summary

**Figure:**
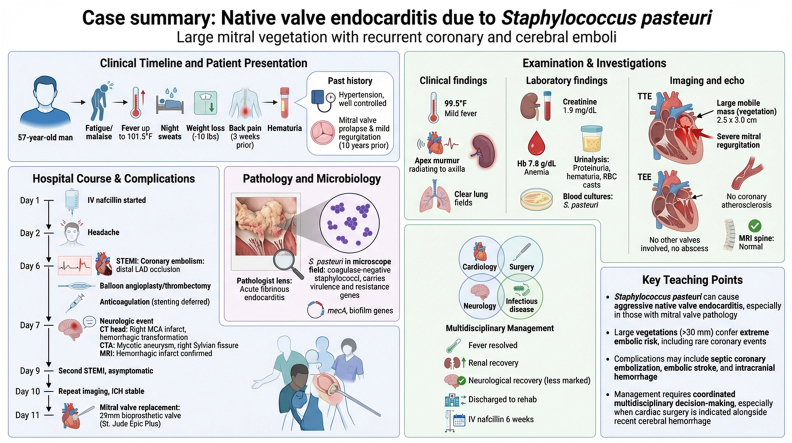

**Visual Summary:**
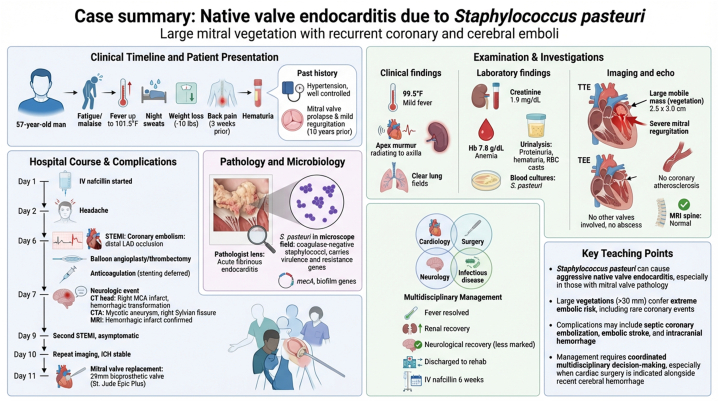
Recurrent Embolic Events and Multidisciplinary Management in *Staphylococcus pasteuri* Infective Endocarditis CT = computed tomography; CTA = computed tomography angiography; ICH = intracranial hemorrhage; IV = intravenous; LAD = left anterior descending artery; MCA = middle cerebral artery; MRI = magnetic resonance imaging; STEMI = ST-segment elevation myocardial infarction; TEE = transesophageal echocardiography; TTE = transthoracic echocardiography.

## History of Presenting Illness

A 57-year-old man presented with a 2-month history of progressive fatigue, malaise, intermittent fevers up to 101.5 °F, drenching night sweats, and an unintentional 10-pound weight loss. Three weeks before the presentation, he developed persistent lower back pain treated with nonsteroidal anti-inflammatory drugs at an urgent care. One week before admission, he noted 2 episodes of gross hematuria without dysuria. He denied recent dental procedures, intravenous drug use, travel, or animal exposure.

**Take-Home Messages**

• *Staphylococcus pasteuri* is a virulent pathogen capable of causing significant endocardial destruction and should not be dismissed as a contaminant.
• Large vegetations associated with this organism carry an exceptionally high risk for recurrent embolic events, including rare coronary septic embolization.
• Early multidisciplinary consultation is vital to navigate the timing of surgery when embolic risk and intracranial hemorrhage coexist.

Vitals were notable for a temperature of 99.5 °F. Cardiovascular examination revealed a regular rhythm and a prominent grade 3/6 holosystolic murmur at the apex, radiating to the axilla, which was a significant change from the mild murmur noted in his historical records. Lung auscultation demonstrated clear breath sounds bilaterally without crackles or wheezes. His abdomen was soft and nontender, although mild splenomegaly was noted upon deep palpation. Skin examination was negative for stigmata of infective endocarditis (IE). Neurologic examination was initially unremarkable, with no focal motor or sensory deficits.

## Past Medical History

The patient's medical history was notable for essential hypertension, which was well controlled with lisinopril. Ten years before this admission, during a routine physical examination, a systolic murmur was noted. A subsequent transthoracic echocardiogram led to a diagnosis of mitral valve prolapse with mild mitral regurgitation (MR), which was followed up with routine surveillance.

## Investigations

Laboratory evaluation demonstrated a serum creatinine level of 1.9 mg/dL (baseline 1.0 mg/dL) and a hemoglobin level of 7.8 g/dL. Urinalysis revealed 2+ proteinuria, 3+ hematuria, and red blood cell casts consistent with glomerulonephritis. Urine culture was negative, excluding a urinary source of primary infection. Two sets of blood cultures grew *Staphylococcus pasteuri*. The admission electrocardiogram (ECG) showed a normal sinus rhythm (Figure 1).

**Figure 1:**
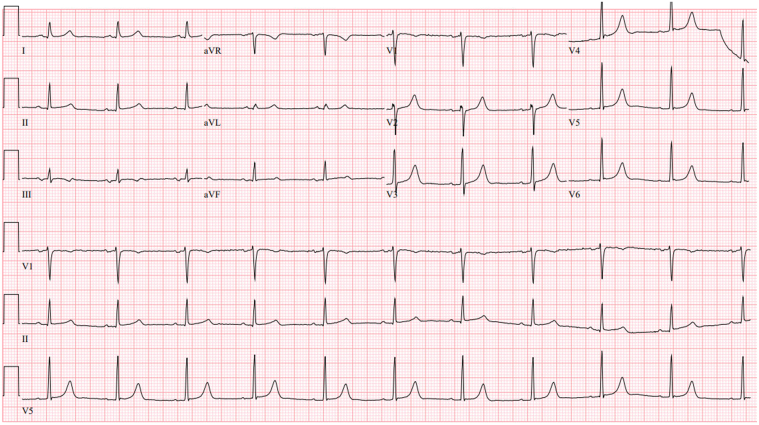
Electrocardiogram on Admission With Normal Sinus Rhythm

Transthoracic echocardiography demonstrated a left ventricular ejection fraction of 60%, a dilated left atrium, a large mobile vegetation on the posterior mitral leaflet, and severe MR (Figure 2). Subsequent transesophageal echocardiography confirmed a 2.5 × 3.0 cm friable, mobile vegetation on the atrial aspect of the mitral valve extending onto the subvalvular apparatus with flail posterior leaflet and severe anteriorly directed MR (Videos 1 and 2, Figure 3). No abscess or involvement of other valves was identified. Preoperative coronary angiography demonstrated no obstructive coronary artery disease (Video 3, Figure 4). Magnetic resonance imaging of the spine, obtained to evaluate back pain, showed no evidence of osteomyelitis or discitis.

**Figure 2:**
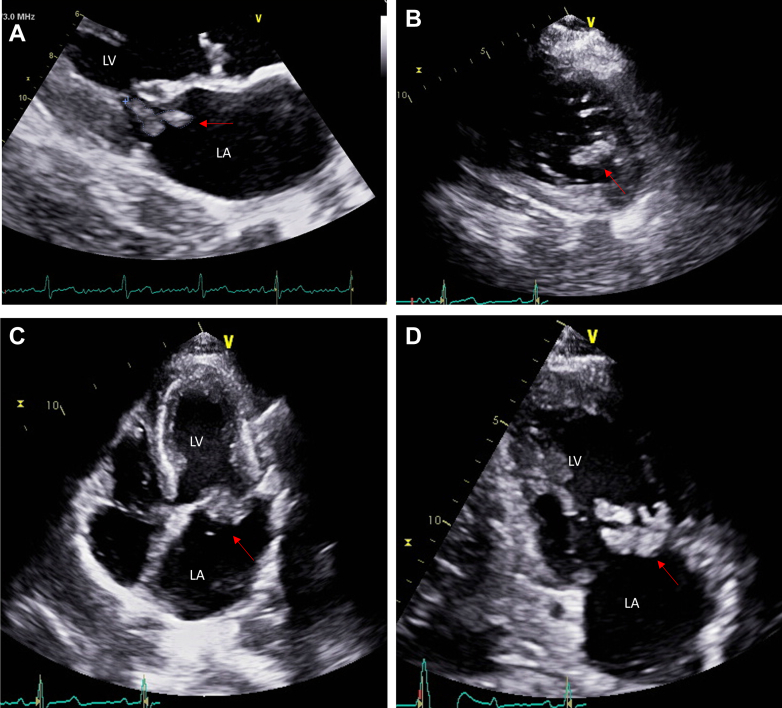
Findings on Transthoracic Echocardiography Transthoracic echocardiography with (A) zoomed-in parasternal long-axis view, (B) short-axis view of the mitral valve, (C) apical 4-chamber view, and (D) apical 2-chamber view showing a large 3-cm multilobulated echodensity on the posterior leaflet of the mitral valve (red arrow). LA = left atrium; LV = left ventricle.

**Figure 3:**
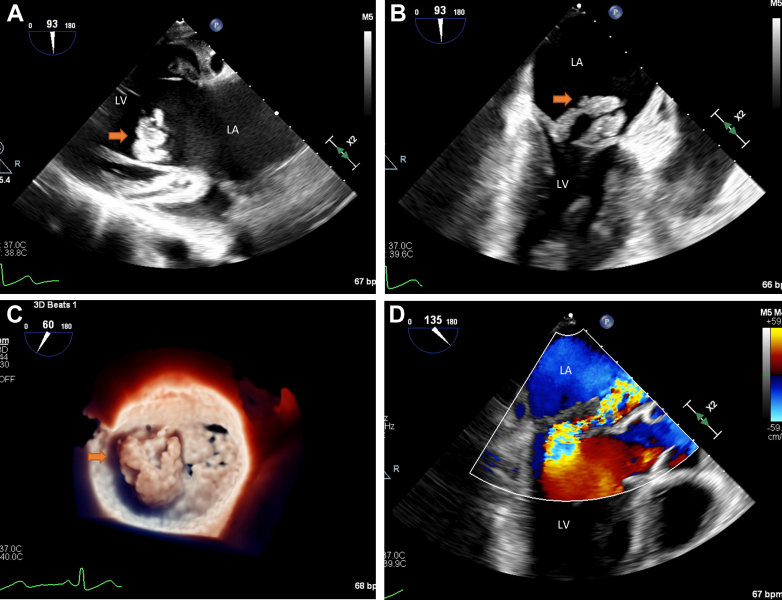
Transesophageal Echocardiography of the Mitral Valve Transesophageal echocardiogram showing (A) transgastric long-axis view of the mitral valve at approximately 90° with a large vegetation attached to the posterior mitral leaflet (orange arrow); (B) mid-esophageal view of the mitral valve at 90° showing a large 2.5 × 3.0 cm vegetation on the atrial aspect of the mitral valve extending onto the subvalvular apparatus with a flail posterior leaflet (orange arrow); (C) 3D bicommissural view of the mitral valve with a large vegetation attached to the posterior leaflet (orange arrow); and (D) mid-esophageal long-axis view of the mitral valve at 135° with severe eccentric and anteriorly directed mitral regurgitation. 3D = 3-dimensional; LA = left atrium; LV = left ventricle.

**Figure 4:**
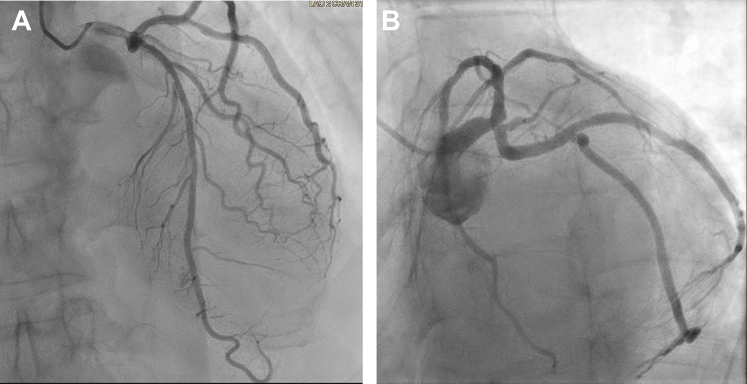
Findings on Preoperative Coronary Angiogram on Day 3 Coronary angiogram with (A) AP Cranial view showing mild LAD disease (B) LAO Caudal view showing moderate distal Left Main disease. AP = anteroposterior; LAO = left anterior oblique.

## Management

Intravenous nafcillin was initiated per infectious diseases consultation. On hospital day 2, the patient reported a new headache that resolved with analgesics. Given the echocardiographic findings, early mitral valve replacement was planned pending blood culture clearance.

On hospital day 6, ST-segment elevation was identified on telemetry monitoring. The patient denied chest pain. A 12-lead ECG confirmed anterolateral ST-segment elevation myocardial infarction (Figure 5). Emergent coronary angiography revealed complete occlusion of the distal left anterior descending artery (Video 4). Given the clinical context of large mitral vegetation and active bacteremia, septic coronary embolization was diagnosed. Mechanical thrombectomy with balloon angioplasty was performed; stenting was deferred given the embolic etiology and anticipated surgery. Intravenous anticoagulation with heparin was initiated (Figure 6).

**Figure 5:**
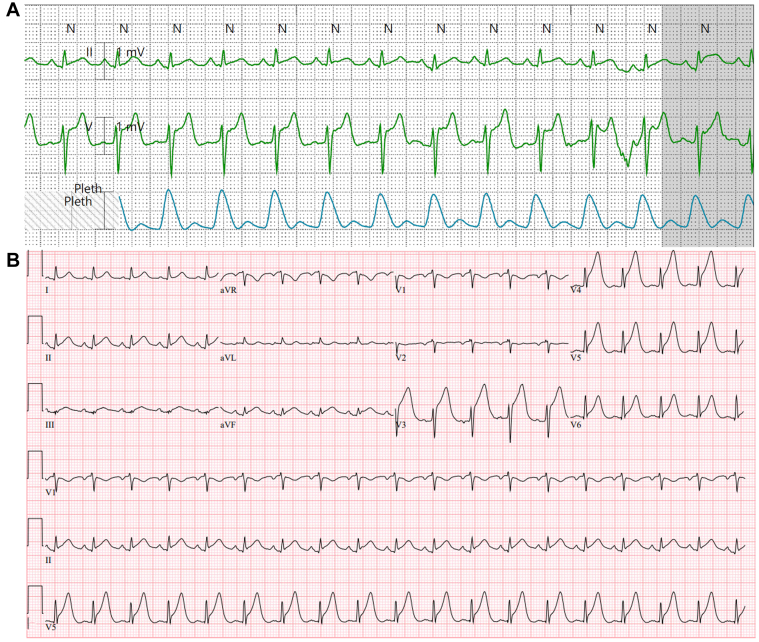
Findings During Asymptomatic STEMI on Day 6 (A) Telemetry strip and (B) electrocardiogram on day 6 showing ST-segment elevation in anterolateral leads.

**Figure 6:**
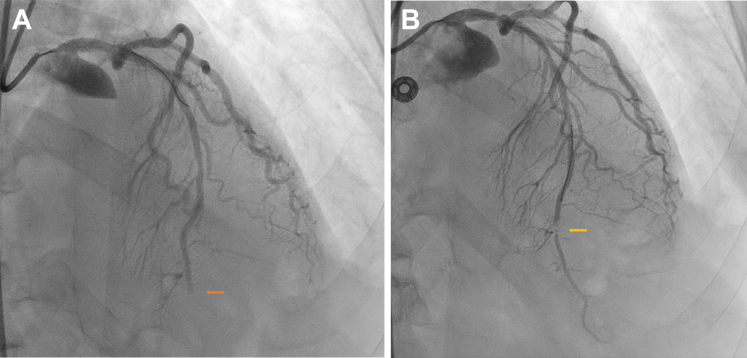
Coronary Angiography on Day 6 Coronary angiogram showing (A) acute thrombotic occlusion of the distal left anterior descending artery (orange arrow) and (B) post–mechanical thrombectomy and balloon angioplasty with a focal haziness (yellow arrow) and TIMI flow grade 3.

On hospital day 7, the patient developed acute right-sided facial droop and expressive aphasia. Noncontrast computed tomography of the head revealed a mixed-density process in the right frontal lobe and adjacent right lateral basal ganglia and insular cortex, concerning for ischemic infarction with cortical and subarachnoid hemorrhagic transformation. Computed tomography angiography of the head and neck demonstrated attenuation of the M2 branches of the right middle cerebral artery (MCA) and a 2-mm focus of contrast enhancement in the right sylvian fissure consistent with a mycotic aneurysm. Brain magnetic resonance imaging confirmed subacute right MCA territory infarction with hemorrhagic transformation (Figure 7). Surgical intervention was deferred pending neurologic stabilization as recommended by neurology and neurosurgery.

**Figure 7:**
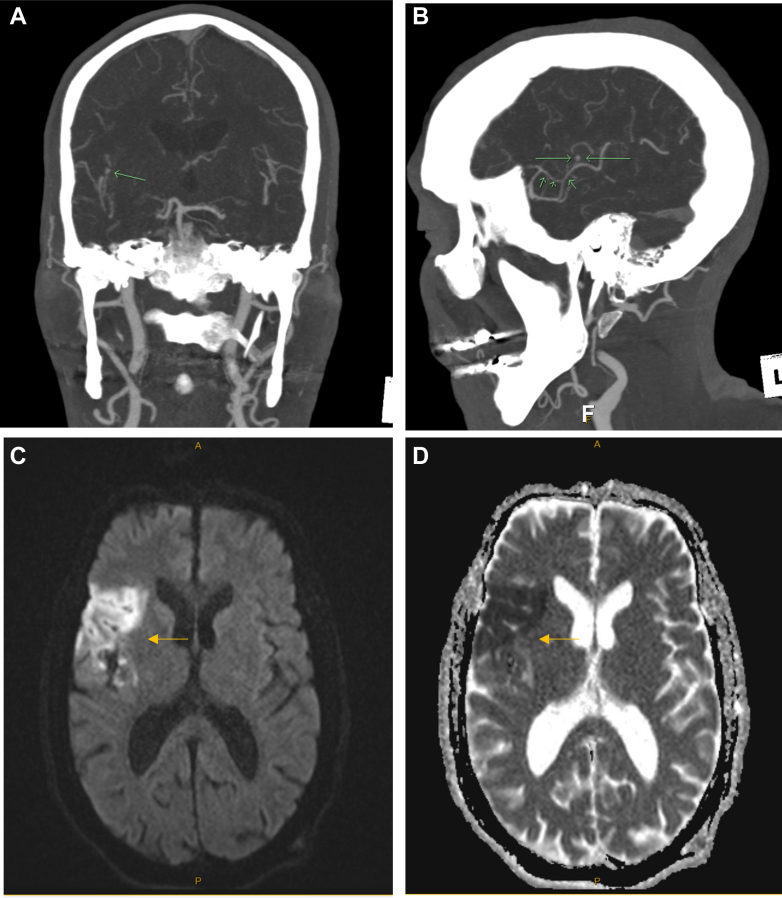
Intracranial Imaging During Acute Stroke on Day 7 (A and B) Computed tomography angiography head and neck showing a rounded 2-mm focus of enhancement concerning for the aneurysm of the right M2 sylvian branch (green arrow). (C and D) Diffusion weighted images of magnetic resonance imaging brain showing subacute right middle cerebral artery territory infarction with hemorrhagic transformation (yellow arrow).

On hospital day 9, a second, asymptomatic ST-segment elevation was detected on telemetry, and confirmed on ECG. Repeat coronary angiography revealed a distal left anterior descending artery occlusion consistent with recurrent septic embolization; no intervention was performed given the distal location, hemodynamic stability, and lack of symptoms (Video 5, Figure 8).

**Figure 8:**
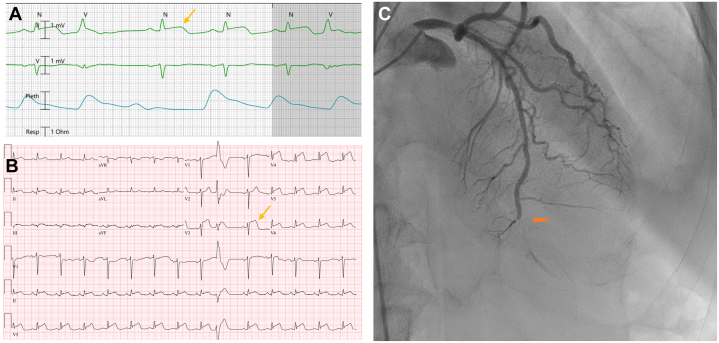
Findings During Second Asymptomatic STEMI on Day 9 (A) Telemetry strip with ST-segment elevation (yellow arrow). (B) Electrocardiogram with ST-segment elevation (yellow arrow) in the anterior leads with premature ventricular complexes. (C) Coronary angiogram showing acute occlusion of the distal left anterior descending artery (red arrow).

On hospital day 10, repeat cranial imaging confirmed stability of the intracranial hemorrhage (ICH). The following day, the patient underwent successful mitral valve replacement with a 29-mm St Jude Epic Plus bioprosthetic valve via median sternotomy on cardiopulmonary bypass. Histopathologic examination of the excised valve confirmed acute fibrinous endocarditis.

## Follow-Up

Fevers resolved within 48 hours postoperatively. Renal function improved progressively, with the serum creatinine level returning to 1.2 mg/dL at discharge. Expressive aphasia largely resolved, with residual mild right-sided facial droop, and the patient was discharged to an inpatient rehabilitation facility to complete a 6-week course of intravenous nafcillin.

On outpatient neurosurgical follow-up, interval surveillance imaging identified a new left MCA mycotic aneurysm. Cerebral angiography was performed, and the aneurysm was managed conservatively given its size and stability. The patient subsequently completed cardiac rehabilitation and has remained clinically well.

## Discussion

First described in 1993, *S pasteuri* is a coagulase-negative staphylococcus (CoNS) historically misidentified as *Staphylococcus warneri* due to phenotypic similarities.^1^ While CoNS are typically associated with prosthetic valves or indwelling devices, *S pasteuri* is increasingly recognized as a primary pathogen in native valve endocarditis (NVE).^2^ Genomic analysis has identified virulence factors including biofilm-associated genes and the *mecA* gene, which confer adherence, antibiotic tolerance, and methicillin resistance, features that likely contributed to the substantial vegetation size and subvalvular destruction observed in this case.^3^

The clinical course of *S pasteuri* endocarditis is typically subacute, characterized by fever, constitutional symptoms, and progressive valvular dysfunction. Although CoNS account for approximately 8% of NVE cases in large multicenter studies, literature specific to *S pasteuri* remains sparse, often leading to underestimation of its severity. While some reports in immunocompromised hosts describe an indolent course, our case aligns with observations by Sabe et al,^4^ suggesting that in patients with preexisting valvular pathology, *S pasteuri* can exhibit a virulence profile comparable to *Staphylococcus aureus*.^5^

The present case is distinguished from prior reported cases by 3 features: an exceptionally large, friable vegetation; recurrent septic coronary embolization, itself a rare complication occurring in fewer than 1% of IE cases^6^; and the concurrent presence of mycotic aneurysm, embolic stroke, and ICH, which collectively created a formidable therapeutic dilemma. Notably, no definitive portal of entry was identified despite thorough clinical evaluation, a pattern consistent with prior reports of *S pasteuri* bacteremia arising in the absence of an obvious source, particularly in the setting of underlying valvular pathology.

Systemic embolization complicates 20% to 50% of IE cases, with risk correlating directly with vegetation size and mobility.^7^^,^^8^ Vegetations exceeding 1 cm carry high embolic risk; at 3 cm, the vegetation in this patient represented an extreme embolic threat. Recurrent septic coronary embolization within 72 hours is particularly exceptional given that coronary involvement complicates fewer than 1% of IE cases, and further underscores the aggressive embolic potential of large, mobile vegetations in *S pasteuri* NVE.

Management of *S pasteuri* IE is guided by broader staphylococcal principles given the absence of species-specific data. The concurrent ICH introduced a critical surgical timing dilemma: while current guidelines recommend delaying cardiac surgery following ICH to minimize perioperative neurologic risk, the persistent embolic burden, evidenced by a second coronary event and an evolving mycotic aneurysm, necessitated early intervention. Following multidisciplinary consensus among cardiology, cardiothoracic surgery, neurology, neurosurgery, and infectious diseases, surgery was performed after confirmation of ICH stability. A bioprosthetic valve was selected to avoid the requirement for long-term anticoagulation, which remained contraindicated in the setting of recent hemorrhage.

## Conclusions

This case illustrates the capacity of *S pasteuri* to cause severe NVE with extensive valvular destruction and high systemic embolic burden. Large, friable vegetations carry substantial risk for recurrent embolization, including rare but life-threatening coronary involvement. When surgical indications coexist with ICH, management demands individualized, multidisciplinary decision-making to optimize both cardiac and neurologic outcomes.

## Funding Support and Author Disclosures

The authors have reported that they have no relationships relevant to the contents of this paper to disclose.

## Appendix

Video 1 Transesophageal Echocardiography ME View of the Mitral Valve at Approximately 130° Showing a Large Vegetation Attached to a Flail Posterior Mitral Valve Associated With Resultant Severe Anteriorly Directed Mitral Regurgitation

Video 2 Transesophageal Echocardiography Mid Esophageal View of the Mitral Valve at Approximately 130° Showing a Large Vegetation Attached to a Flail Posterior Mitral Valve Associated With Resultant Severe Anteriorly Directed Mitral Regurgitation

Video 3 Initial Preoperative Coronary Angiogram Showing Moderate Distal Left Main Disease and Mild Mid–Left Anterior Descending Artery Disease

Video 4 Coronary Angiogram Demonstrating Acute Thrombotic Occlusion of the Distal Left Anterior Descending Artery on Day 6

Video 5 Repeat Coronary Angiogram Showing Thrombotic Occlusion of the Distal Left Anterior Descending Artery on Day 9
